# Isolating adverse effects of glucocorticoids on the embryonic cardiovascular system

**DOI:** 10.1096/fj.202000697R

**Published:** 2020-06-05

**Authors:** Noor E. W. D. Teulings, Tessa A. C. Garrud, Youguo Niu, Katie L. Skeffington, Christian Beck, Nozomi Itani, Fiona G. Conlon, Kimberley J. Botting, Lisa M. Nicholas, Thomas J. Ashmore, Heather L. Blackmore, Wen Tong, Emily J. Camm, Jan B. Derks, Angela Logan, Michael P. Murphy, Susan E. Ozanne, Dino A. Giussani

**Affiliations:** 1 Institute of Metabolic Science-Metabolic Research Laboratories, University of Cambridge, Addenbrooke’s Hospital, Cambridge, UK; 2 Department of Physiology, Development and Neuroscience, University of Cambridge, Cambridge, UK; 3 Department of Perinatal Medicine, University Medical Centre, Utrecht, Netherlands; 4 Medical Research Council Mitochondrial Biology Unit, University of Cambridge, Cambridge, UK; 5 Department of Medicine, University of Cambridge, Cambridge, UK

**Keywords:** antenatal glucocorticoid therapy, cardiac function, cellular senescence, oxidative stress

## Abstract

Antenatal glucocorticoid therapy reduces mortality in the preterm infant, but evidence suggests off-target adverse effects on the developing cardiovascular system. Whether deleterious effects are direct on the offspring or secondary to alterations in uteroplacental physiology is unclear. Here, we isolated direct effects of glucocorticoids using the chicken embryo, a model system in which the effects on the developing heart and circulation of therapy can be investigated, independent of effects on the mother and/or the placenta. Fertilized chicken eggs were incubated and divided randomly into control (C) or dexamethasone (Dex) treatment at day 14 out of the 21-day incubation period. Combining functional experiments at the isolated organ, cellular and molecular levels, embryos were then studied close to term. Chicken embryos exposed to dexamethasone were growth restricted and showed systolic and diastolic dysfunction, with an increase in cardiomyocyte volume but decreased cardiomyocyte nuclear density in the left ventricle. Underlying mechanisms included a premature switch from tissue accretion to differentiation, increased oxidative stress, and activated signaling of cellular senescence. These findings, therefore, demonstrate that dexamethasone treatment can have direct detrimental off-target effects on the cardiovascular system in the developing embryo, which are independent of effects on the mother and/or placenta.

## 1 Introduction

Antenatal Corticosteroid Therapy (ACT) given to women threatened with preterm birth is one of the best examples of the successful translation of basic experimental science into human clinical practice, as it diminishes neonatal morbidity and mortality by accelerating fetal lung maturation.^1,2^ Toward term, the fetal plasma glucocorticoid concentration rises, triggering maturation of vital organ systems in preparation for postnatal life.^3^ Preterm birth (often classified as <37 weeks gestation in humans) occurs prior to this physiological rise in fetal plasma cortisol. The exogenous administration of synthetic corticosteroids is an attempt to mimic the endogenous maturational processes that would otherwise be lacking in the preterm infant.

Despite the overwhelmingly clear life-saving benefits of ACT on preterm babies, there is increasing awareness of adverse off-target effects on organ systems other than the fetal lungs.^4^ For instance, in the cardiovascular system, data derived from human studies as well as from animal models, show evidence of aortic stiffness, endothelial dysfunction, increased cardiac weight, and hypertension in offspring from mothers treated with ACT.^4–8^ Such unwanted side-effects of ACT will have important implications for the long-term health of any baby born preterm as well as offspring from ~50% of women who have received ACT but actually go on to deliver at term.^9^

Since glucocorticoids are administered to the mother in pregnancy, it is unclear whether any adverse effects on the cardiovascular system of the offspring following ACT are a consequence of direct effects on the fetus or secondary to alterations in the physiology of the mother and/or the placenta. For instance, treatment of pregnant ewes with a human clinically relevant dose regimen of ACT reduced fetal growth, while direct treatment of fetal sheep did not,^10^ implying an effect on placental perfusion. Accordingly, Jellyman et al^11^ reported that human clinically relevant doses of ACT in pregnant sheep increased uteroplacental vascular resistance. Administration of glucocorticoids to the mother during late gestation reduces placental weight in all species studied to date.^8,12^ Furthermore, it is known that increased cardiac load stimulates cardiomyocyte growth and proliferation, and that the resistance of the placental vasculature can regulate fetal cardiac maturation.^13^ This again indicates possible effects of ACT on the fetal cardiovascular system secondary to those at the level of the placenta.

To improve current clinical ACT and make it safer for preterm babies, it is therefore important to understand any direct vs indirect effects of treatment on the developing offspring. In this study, the direct effect of synthetic steroids used in human clinical practice were investigated in the chicken embryo. This is the only established animal model system in which effects on the fetal heart and circulation of therapy can be isolated, independent of effects on the mother and/or the placenta.^14,15^ Of added value, the temporal profile of developmental milestones of the cardiovascular system is similar between humans and chickens.^15^

Therefore, adopting an integrative approach, combining experiments at the isolated organ, cellular and subcellular levels, the aim of this study was to isolate the direct effects of dexamethasone (Dex) on the developing cardiovascular system. The study tested the hypothesis that treatment of the chicken embryo with dexamethasone will have direct adverse effects on the developing cardiovascular system. Underlying mechanisms investigated included alterations in cardiac systolic and diastolic function, cardiac responsiveness to autonomic receptor agonists, cardiomyocyte size and number, generation of oxidative stress, and activation of signaling pathways mediating cardiomyocyte senescence. Treatment of chicken embryos with dexamethasone started at day 14 out of the 21-day incubation period, equivalent to *ca*. 27 weeks of gestation in human pregnancy, a common time in gestation when ACT would be performed. Outcome studies were performed at day 19.

## 2 Methods

### 2.1 Data, materials, and code disclosure statement

The data that support the findings of this study are within the manuscript or available from the corresponding author upon reasonable request.

### 2.2 Ethical approval

All animal procedures conform with the guidelines from Directive 2010/63/EU of the European Parliament on the protection of animals used for scientific purposes. This research was approved under the Animals (Scientific Procedures) Act 1986 Amendment Regulations 2012 following ethical review by the University of Cambridge Animal Welfare and Ethical Review Board (AWERB).

### 2.3 Experimental animals

Several cohorts of fertilized eggs were used for the different analyses. All cohorts were studied contemporaneously within the same experimental season. Fertilized eggs were purchased from Medeggs (Henry Stewart & Co., Norfolk, UK). This supplier delivers fertilized eggs in batches from a single day’s laying, meaning anyone egg used in the present program of work came from a different hen. Egg storage is commonly practiced in the artificial incubation of domestic birds. If the storage temperature for freshly laid chicken eggs is kept below the physiological zero (25-27°C), dormancy of the embryo can be maintained, and fertile eggs can be stored for 1-3 weeks. Eggs from any one cohort were transported, accumulated, and maintained over 3-5 days at 14°C to arrest and synchronize development, prior to incubation.^14^ Fertilized eggs were then incubated under controlled conditions (38°C, 45% humidity, 12:12 hours light: dark cycle automatically rotated every hour using Model 75-A and Model M-240, Masalles, Barcelona, Spain). Embryos were randomized into two groups: control (C) and dexamethasone (Dex). Dexamethasone (dexamethasone 21-phosphate disodium, Sigma-Aldrich) was administered topically onto the chorioallantoic membrane via a small hole in the shell air cell, which could be covered by a small piece of tape post-administration. These procedures were performed by the same person for all cohorts and all scientists measuring outcomes were blinded to treatments. A dose of 0.1 mg/kg, was administered on day 14 of the 21-day total incubation period. The control group received an equal volume of water (0.1 mL). To determine the amount of dexamethasone needed, a pilot study was conducted where the weight of 5 day-14 embryos was established. The mean weight of these five embryos was then used to calculate the dose of 0.1 mg/kg dexamethasone per embryo weight. The dose of 0.1 mg/kg used was based on our previous work in fetal sheep^16,17^ and available human clinical information. Pregnant women threatened with preterm labor receive 24 mg of dexamethasone over a 48 hours period.^1^ Approximately 30% of maternal synthetic glucocorticoids cross the human placenta.^18^ Assuming an average woman weighs 70 kg, ca. 0.1 mg/kg dexamethasone will affect the human fetus.

### 2.4 Biometry

On day 19 of incubation, 30 chicks (n = 15 controls; n = 15 dexamethasone) were humanely killed by dislocation of the neck. Embryos were removed from the eggshell, blotted dried, then weighed and measured. The weight of the embryo was expressed as a percentage of the egg weight on day 19 to determine how much resource was turned into fetal body mass. The heart, brain, and liver were dissected and weighed. Head diameter was measured using a high precision electronic caliper (Mitutoyo 500-181-20, UK), placing the ends just behind the eyes.

### 2.5 Langendorff preparation

In another cohort of 24 embryos (n = 12 controls; n = 12 dexamethasone), after death, the heart was quickly excised and placed in ice-cold Krebs-Henseleit bicarbonate buffer containing sodium chloride (NaCl), potassium chloride (KCl), magnesium sulfate (MgSO_2_)·7H_2_O, potassium dihydrogen sulfate (KH_2_PO_4_), sodium bicarbonate (NaHCO_3_), glucose, and calcium chloride dihydrate (CaCl_2_·2H_2_O) (mmol^−1^). The heart was mounted onto the Langendorff apparatus, cannulated via the aorta and perfused through the coronary arteries with pre-warmed (37°C) and pre-gassed (95% O_2_ and 5% CO_2_) Krebs-Henseleit solution. Isolated hearts were studied in Langendorff mode and perfused at a constant pressure of 35 mmHg. A small, nonelastic balloon was inserted through the left atrium into the left ventricle and gradually filled with 30 μL saline. After stabilization for at least 15 minutes, basal cardiac function was recorded, consisting of heart rate (HR), left ventricular systolic pressure (LVSP), and left ventricular end diastolic pressure (LVEDP). Left ventricular developed pressure (LVDP) was calculated as LVSP-LVEDP. The cardiac responsiveness to the muscarinic receptor agonist Carbachol and the beta 1 (β_1_)-adrenergic agonist Isoprenaline was tested by administering increasing doses of drugs (Carbachol 10^−8^ to 10^−6^ M, Isoprenaline 10^−9^ to 10^−7^ M), randomized for dose order. The drug was washed out and the heart could stabilize at baseline before administration of the next bolus. Responses to Carbachol and Isoprenaline were calculated as the change in values directly before drug administration compared to values following administration.

### 2.6 Cardiac stereology

Another cohort of 20 chicken embryos (n = 10 controls; n = 10 dexamethasone) was heavily anesthetized (0.1 mL intraperitoneal Pentobarbitone Sodium 20%, National Veterinary Services Limited) and perfused intracardially with heparinized saline followed by 4% of paraformaldehyde (PFA) at a physiological pressure of 2.66 kPa, after which the heart and aorta were harvested. The ventricles were cleared of the atria and vessels and sliced at 1 mm thickness with a heart slicer (Zivic Instruments, Pittsburgh, PA, USA). The cardiac slices were imaged with a Canon EOS 5D, macro 50 mm lens (aperture f/6.7, exposure 1/125 seconds), and analyzed using ImageJ 1.48v. A point grid was superimposed on the cardiac sections and mid cardiac areas of left and right ventricles and atria were determined (point/4.00 mm^2^) using the Cavalieri principle.^19^ The mid cardiac section was selected for analysis. The thickness of the wall and the width of the lumen were determined at 10× magnification, using intersection of grid lines (thickness) or spread points (area) placed over the tissue (198 μm apart). The outside of the wall was used as starting point, with lines drawn perpendicular to the overlying grid. An average of 12 measurements were taken per embryo.

A 5 mm segment of the descending aorta at the level of the apex of the heart was embedded in paraffin according to standardized procedures. Sections were cut at 5 μm thickness and mounted onto glass slides per 10 (consecutive) sections. One of these slides was randomly selected and stained with hematoxylin and eosin. Quantification was performed using an Olympus BX-50 microscope and the Computer-Assisted Stereological Toolbox (CAST version 2.0, Olympus, Albertslund, Denmark), with slides blinded for the treatment group.

### 2.7 Fibrosis staining

Another cohort of 13 chicken embryos (n = 5 controls; n = 3 dexamethasone) was perfusion fixed under anesthesia as above and hearts were embedded in paraffin. A 5 μm paraffin embedded slide was cut from whole fixed hearts. Sections were deparaffinized (Histoclear 2 × 5 minutes) and rehydrated in three changes of ethanol (100%, 90%, 70%; each 3 minutes). They were stained with Picrosirius red for 1 hour before being washed in two change of acidified water (5%). After dehydration in three changes of ethanol (70%, 90%, 100%; each 30 seconds), they were dipped in xylene before being cover slipped. Between 4 and 6 pictures per animal were randomly taken with the NewCast system at 40× magnification. These images were analyzed using ImageJ 1.48 software to estimate the percentage of fibrosis by putting a color threshold over pictures of the stained slides.

### 2.8 Cardiomyocyte cell size

In another cohort of 12 chicken embryos (n = 6 controls; n = 6 dexamethasone), after death, individual cardiomyocytes were isolated via enzymatic degradation using the isolated Langendorff preparation. The coronary vasculature was retrogradely perfused via the aorta with Tyrode’s solution (140 mM NaCl, 5 mM KCl, 1 mM magnesium chloride (MgCl_2_), 10 mM Glucose, 10 mM a zwitterionic organic chemical buffering agent (HEPES), pH 7.35) for several minutes until blanched. Enzymes were then added to the circulation for 10 minutes to degrade the cardiovascular tissue. Enzymes used were type II collagenase (160 U/mL, Worthington) and 0.78 U/mL protease type XIV from *Strep. griseus* (Sigma). The heart was then perfused with Kreb’s buffer (50 mM L-Glutamic Acid, 30 mM KCl, 20 mM Taurine, 0.5 mM EGTA, 10 mM HEPES, 10 mM Glucose, 30 mM KH2PO4, 3 mM MgSO4, pH 7.37) for a further 12 minutes to wash out the enzymes. All perfusing solutions were maintained at 37°C. Ventricles were then dissected away and placed into a tube containing roughly 5 mL of Kreb’s buffer and shaken gently to release cells before being allowed to settle for 45 minutes. Following this PFA (10% Sigma) was added to reach a 4% solution. Cell suspensions were stored at room temperature. To assess both nuclearity and cardiomyocyte length or width, solutions were stained with 50% of Methyl Blue stain (Sigma, UK) and visualized under a light microscope (Leica DMI 6000B, UK). The NewCast software (Computer-Assisted Stereological Toolbox, Visiopharm Integrator system version 4.6.3.857) was used to randomly search each 15 μL solution sample to measure 50 randomly chosen cardiomyocytes per heart. Cardiomyocytes were assessed for nuclearity, thickness and length, and cardiomyocyte volume was estimated (see Figure 4).

### 2.9 Determination of numerical cardiomyocyte nuclear density

Another cohort of 12 chicken embryos (n = 6 controls; n = 6 dexamethasone) was heavily anesthetized (0.1 mL intraperitoneal Pentobarbitone Sodium 20%, National Veterinary Services Limited) and perfusion fixed in 4% of formaldehyde, as before. The left ventricle was cut into 1 mm^2^ cubes and these were sorted and selected using the smooth fractionator principle. Tissue cubes were divided into groups of 6-10, with one group embedded in glycolmethacrylate resin. Using a glass blade microtome (2050 Microtome, Reichert-Jung, Munich, Germany), the tissue was cut at 30 μm thickness. To identify cardiomyocyte nuclei, sections were stained with Hematoxylin and 0.15% of Basic Fuchsine (both from Sigma-Aldrich, Gillingham, UK). The numerical density of cardiomyocyte nuclei was established using the Optical dissector technique.^20^ NewCast software (Computer-Assisted Stereological Toolbox, Visiopharm Integrator system software version 4.6.3.857) randomly, systematically, and uniformly assigned a counting frame to each ventricle piece. Point counting was performed using 15 frames, each covering an area of 400 μm^2^, and a dissector height of 10 μm. To determine the numerical density of cardiomyocyte nuclei, the following equation was used:

$$\text{Cardiomyocte}\;\;\text{number}\;\;\text{per}\;\;{\mu \text{m}}^{3}\;\;\text{left}\;\;\text{ventricle}=\frac{\Sigma Q}{h\times \left(ap\right)\Sigma P}$$

where *Q* is the total number of nuclei counted, *h* is the z-height of the dissector, *a/p* is the area of ventricle that each point represents, and Σ*P* is the sum of points that lie over ventricular tissue.

### 2.10 Quantification of mRNA expression using quantitative reverse transcription polymerase chain reaction (qRT-PCR)

In another cohort of 20 chicken embryos (n = 8 controls; n = 12 dexamethasone), after death, whole hearts (left and right ventricle excluding atria) were collected through macro dissection and snap frozen in liquid nitrogen for future analysis. Samples were stored at –80°C for less than 6 weeks prior to experiments. Cardiac ribonucleic acid (RNA) was extracted using the kit that enables purification of total RNA (miRNeasy) Mini Kit 12 (Qiagen, Hilden, Germany). Powdered tissue (80 mg) was homogenized in 700 μL QIAzol Lysis reagent (Qiagen) and a protocol was followed using the manufactures instructions. Negative controls containing no RNA or no reverse transcriptase were used to test for DNA contamination. RNA concentration was quantified using the NanoDrop ND-1000 Spectrophotometer (Thermo Scientific, Waltham, USA). Complementary DNA (cDNA) was generated from 3 μg total RNA per sample by reverse transcription using Thermo Scientific RevertAid First Strand cDNA Synthesis kit (Thermo Scientific, Waltham, Massachusetts, USA). Synthesized cDNA was stored at –20°C prior to the real-time PCR assay. Real-time quantitative PCR was performed using SYBR Green master mix and synthetic primers. A primer mastermix was made containing 0.6 μL of the selected primer, 2.4 μL nuclease free water, and 5 μL SYBR Green per sample. A total of 8 μL mastermix and 2 μL cDNA ([3.75 ng/μL]) was loaded per well. Primers were chosen based on their nucleotide length, location, and self-complementarity and checked for specificity using NCBI nucleotide BLAST. The forward and reverse primer sequences are shown in Table 1. Three housekeepers were tested for this analysis (hypoxanthine phosphoribosyltransferase (HPRT), succinate dehydrogenase complex flavoprotein subunit A (Sdha), and β-Tubulin). These housekeepers were validated through a deletion experiment, from which β-Tubulin appeared to be the least stable, and was therefore, excluded from further analysis. We did not see any influence of glucocorticoid treatment on HPRT or Sdha expression. Therefore, target gene expression was normalized to the geometric mean of HPRT and Sdha. Antenatal glucocorticoid treatment did not statistically influence the expression of these housekeeping genes. Statistics were performed on the relative fold change (2^−ΔΔCT^).

### 2.11 Protein carbonylation assay

Frozen hearts (n = 6 controls; n = 7 dexamethasone; left and right ventricle excluding atria) were homogenized to powder on dry ice using pestle and mortar. Cell lysates were prepared from homogenates using ice-cold cell lysis buffer (1 mL of buffer per 100 mg of tissue; Cell Signaling Technology, UK) containing protease inhibitors (Roche Diagnostics, UK) and protein concentration was determined using a bicinchoninic acid assay (Thermo Fisher Scientific, UK). To determine the extent of posttranslational protein carbonylation that occurs as a result of oxidative damage to proteins, an OxyBlotTM analysis was performed using an OxyBlot detection kit according to the manufacturer’s specifications (Millipore, Billerica, MA). Protein lysates were treated to derivatize carbonyl groups to 2, 4-dinitrophenyl-hydrazone (DNP) moieties. These lysates were then separated on a 12% of SDS-PAGE agarose gel and transferred onto a nitrocellulose membrane (pore size of 0.25 μm; Hybond ECL, Sigma-Aldrich, UK). Nonspecific binding was inhibited by blocking the membrane in 2.5% (w/v) bovine serum albumin (BSA) in tris-buffered saline (TBS) + 0.1% (v/v) Tween 20 (Sigma-Aldrich; tris-buffered saline and polysorbate (TBS-T)) for 1 hour at room temperature. The nitrocellulose membrane was incubated with a primary rabbit anti-DNPH antibody (Chemicon OxyBlot; diluted 1:200) for 1 hour at room temperature. Following incubation with the primary antibody, membranes were washed with TBS-T and incubated with a secondary antibody conjugated with horseradish peroxidase against Rabbit immunoglobulin G (IgG) (Thermo Fisher, UK; diluted 1:10 000) for 1 hour at room temperature. Protein band densities were quantified using ImageJ software (NIH) and normalized against Ponceau S staining, which has been found to be a reliable loading control similar to ß-actin quantification.^21^

### 2.12 Plasma dexamethasone concentrations

In a final group of embryos (n = 6 controls; n = 6 dexamethasone), chorioallantoic fetal blood was taken 24 hours after treatment for measurement of circulation levels of dexamethasone. Plasma concentrations of dexamethasone were determined by high performance liquid chromatography. The sensitivity of the assay was 1-3 ng mL^−1^. The inter- and intraassay coefficients of variation were <10%.

### 2.13 Statistical analysis

Appropriate power calculations derived from previous data sets were performed to determine the minimum sample size required to achieve statistical significance set at *P* < .05. Data were analyzed using GraphPad Prism v5.0. Data are presented as mean ± SEM unless otherwise stated. Groups were statistically compared using Two-way RM ANOVA with Tukey test or the Student’s *t* test for unpaired data, as appropriate. A probability level of 5% (*P* < .05) was taken to be significant.

## 3 Results

### 3.1 Biometry

**Figure 1:**
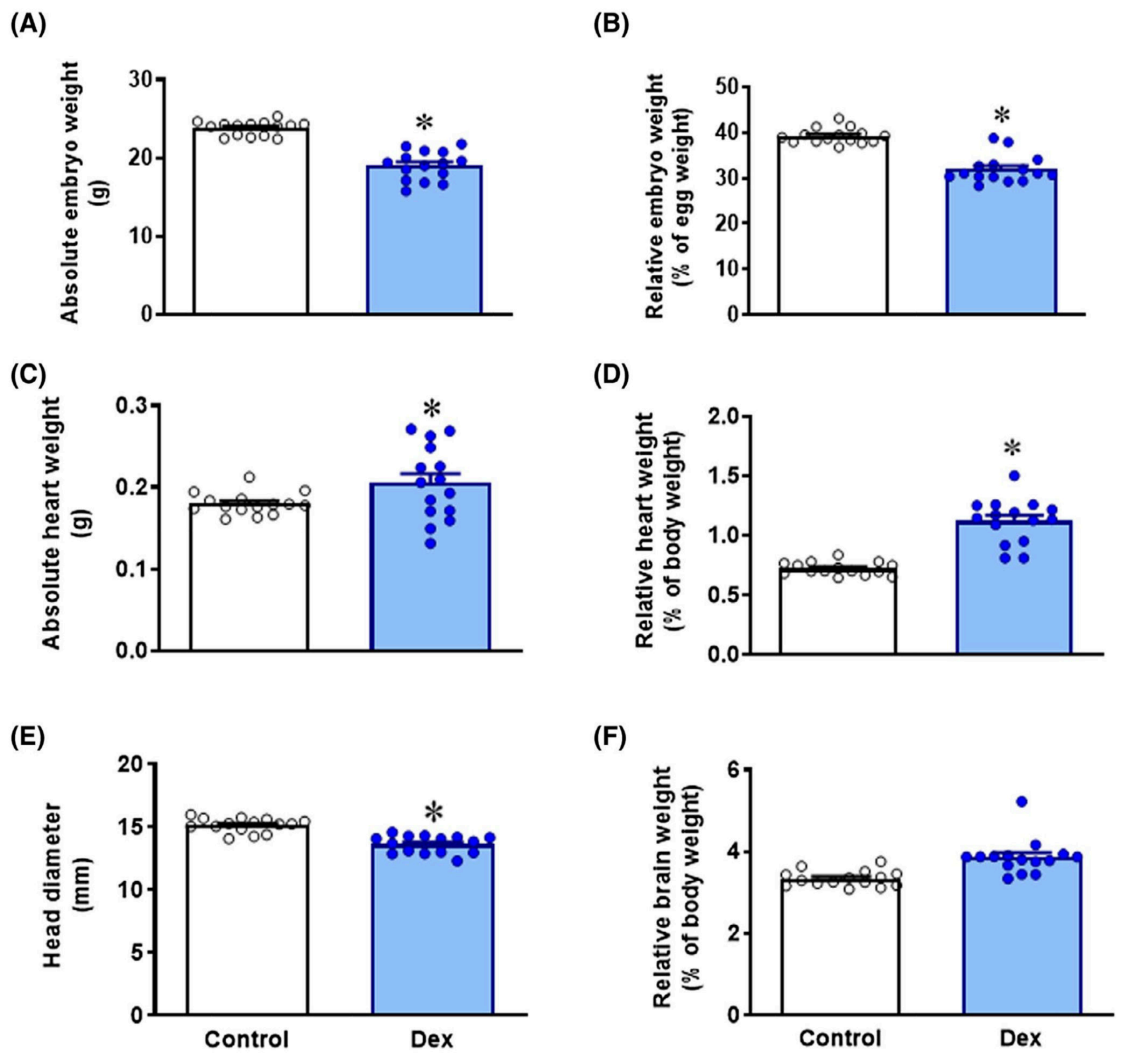
Biometry at 19 days of incubation. Data are mean ± SEM for A, Absolute embryo weight in grams, B, Embryo weight relative to initial egg weight, C, Absolute heart weight in grams, D, Heart weight relative to body weight, E, Head diameter in millimeters, F, Brain weight relative to body weight. Significant difference *P < .05 vs control. Student’s *t* test for unpaired data

Chicken embryos exposed to dexamethasone on day 14 had significantly reduced body weight on day 19, in absolute terms as well as relative to initial egg mass (Figure 1A,B). Chicken embryos treated with dexamethasone had significantly heavier hearts in absolute terms (Figure 1C) and when expressed relative to body weight (Figure 1D). Chicken embryos treated with dexamethasone also had significantly reduced absolute head diameter but when brain weight was expressed relative to body weight, the ratio was significantly increased in dexamethasone-treated embryos compared with controls (Figure 1F). The absolute (0.470 ± 0.01 vs 0.459 ± 0.03, n = 9) and relative (2.12 ± 0.03 vs 2.54 ± 0.18, n = 9) weight of the liver was not different between control and dexamethasone-treated embryos.

### 3.2 Isolated cardiac function

**Figure 2:**
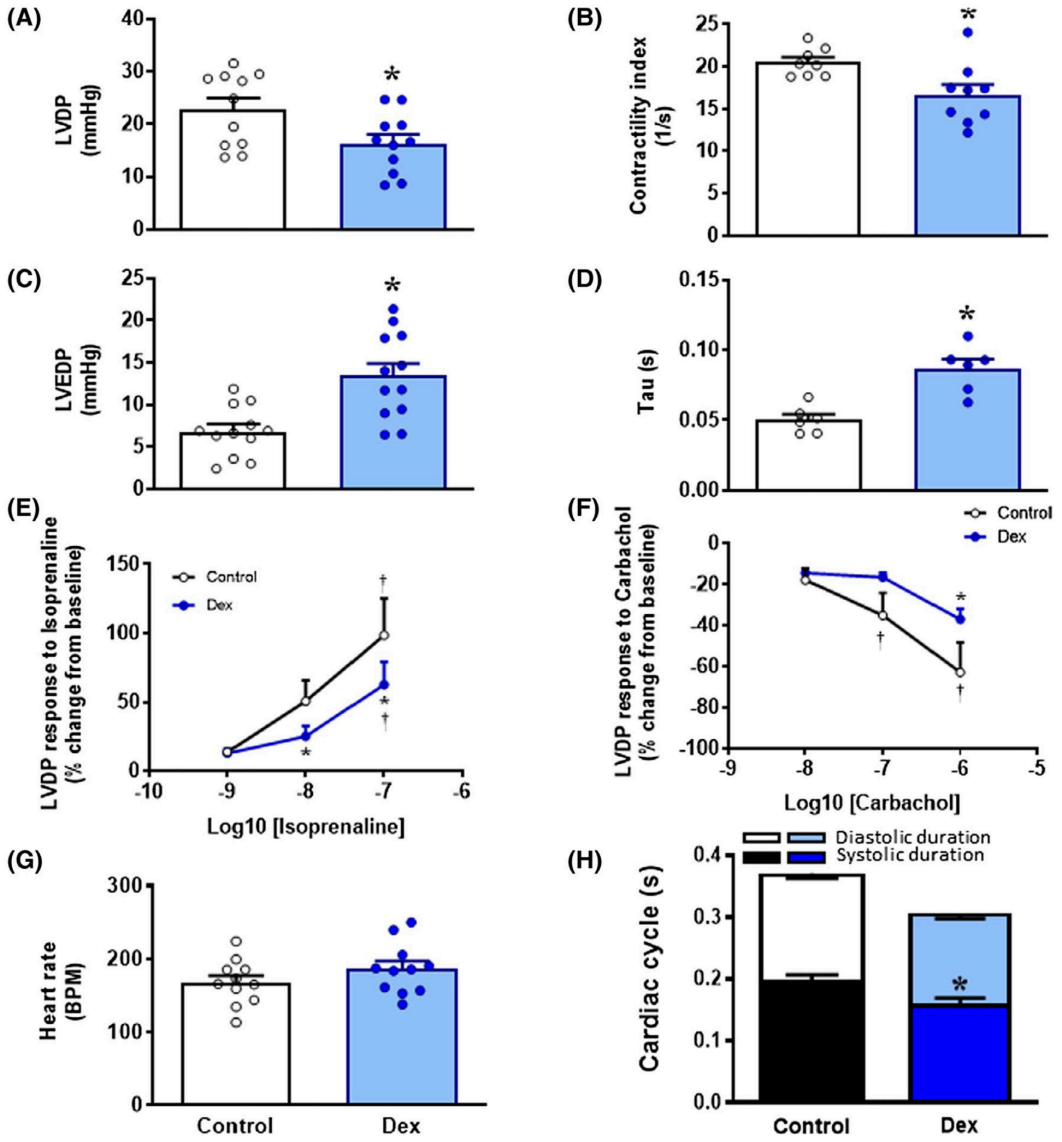
Cardiac function at 19 days of incubation. Data are mean ± SEM for A, Left ventricular developed pressure (LVDP), B, Contractility index, C, Left ventricular end diastolic pressure (LVEDP, D, Left ventricular relaxation time constant (Tau), E & F, Left ventricular inotropic responses to Isoprenaline and Carbachol (E and F; n = 9 controls; n = 8 dexamethasone), G, Heart rate (n = 11 controls; n = 11 dexamethasone), H, Cardiac cycle duration (n = 12 controls; n = 12 dexamethasone). Significant difference *P < .05 vs control, † vs lowest dose. Student’s *t* test for unpaired data for *A-F*, two-way RM ANOVA with Tukey test for E and F

The Langendorff preparation revealed that hearts from dexamethasone-treated embryos had impaired basal systolic and diastolic function, represented by a fall in Left Ventricular Developed Pressure (LVDP) and in the contractility index and an increase in LVEDP and in the time constant Tau, respectively (Figure 2A–D). Relative to controls, changes in LVDP in response to sympathetic and parasympathetic receptor agonists were both diminished in dexamethasone-treated embryos (Figure 2E,F). Although basal HR was unaltered, there was a decrease in the duration of systole, but not diastole, in the cardiac cycle of dexamethasone-treated embryos (Figure 2G,H).

### 3.3 Cardiac stereology, aortic histology, and cardiomyocyte analysis

**Figure 3:**
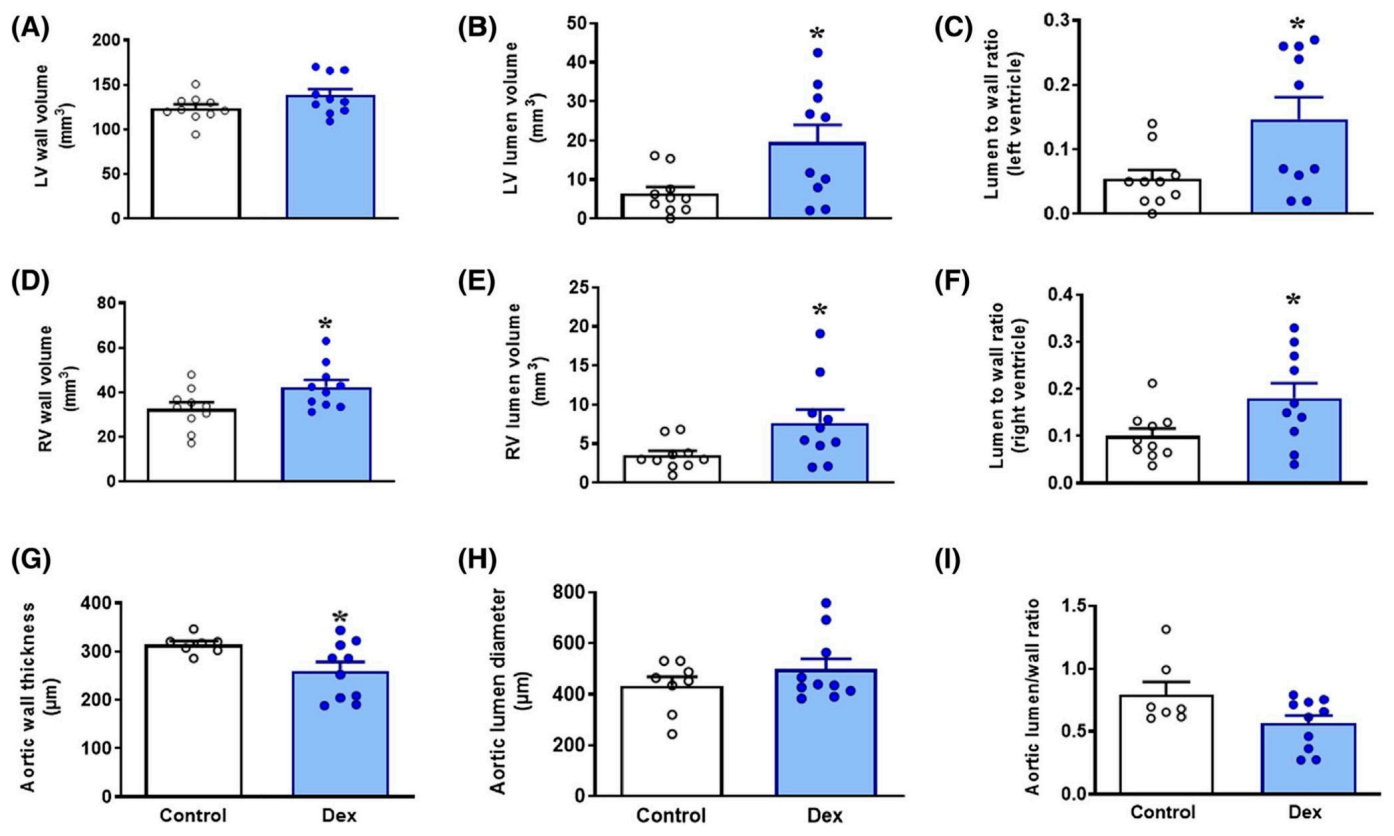
Cardiac and aortic structure at 19 days of incubation. Data are mean ± SEM. A, Left ventricular wall volume in cubic millimeters, B, Left ventricular lumen volume in cubic millimeters, C, Ratio between left ventricular wall and lumen volume, D, Right ventricular wall volume in cubic millimeters, E, Right ventricular lumen volume in cubic millimeters, F, Ratio between right ventricular wall and lumen volume, G, Aortic wall thickness in micrometers, H, Aortic lumen diameter in micrometers, I, Ratio between aortic wall thickness and lumen diameter. Significant difference *P < .05 vs control. Student’s *t* test for unpaired data

**Figure 4:**
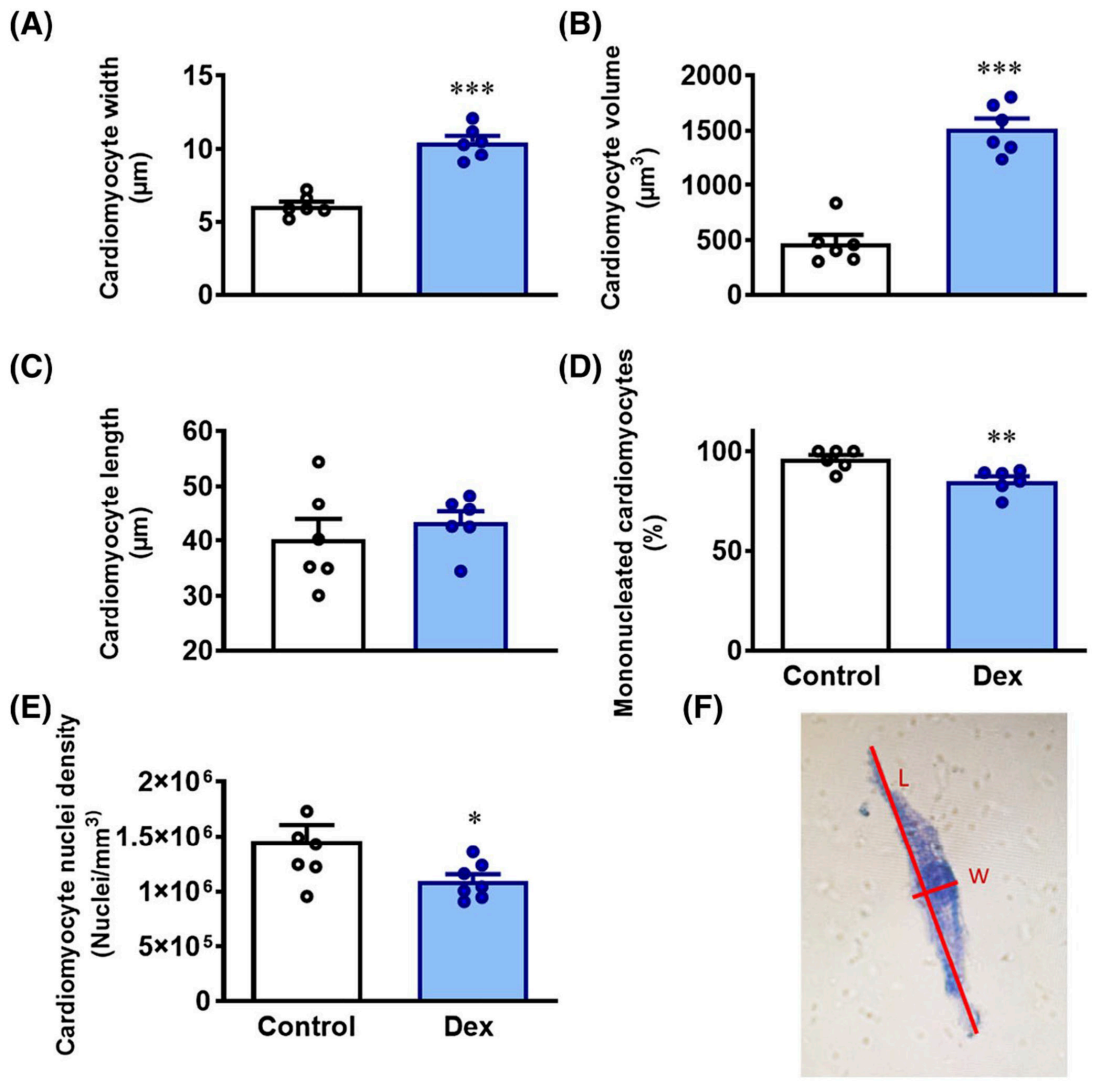
Cardiomyocyte size and nuclearity at 19 days of incubation. Data are mean ± SEM. A, Width of cardiomyocytes in micrometers, B, Cardiomyocyte volume in cubic micrometers, C, Length of cardiomyocytes in micrometers, D, Percentage of mononucleated cardiomyocytes, and E, Cardiomyocyte nuclei density in nuclei per cubic millimeters. Significant difference *P < .05 vs control. Student’s *t* test for unpaired data. Panel F shows a photo of an isolated cardiomyocyte stained with Methyl Blue, illustrating how cardiomyocyte length and width were measured. Width was measured across the level of the nucleus. Length was a measure of the furthest apart edges. Estimated cardiomyocyte volumes were then calculated according to Österman et al^50^ This uses the mathematical approximation of two cones with cone base width equal to cardiomyocyte width, and cone length equal to half of the cardiomyocyte length: $\text{volume}=\frac{2}{3}\pi \frac{lw^{2}}{4}$

Hearts from dexamethasone-treated embryos showed a dilated phenotype (Figure 3A–F), with the left and right ventricular lumen volume significantly increased. Furthermore, aortas from dexamethasone-treated embryos had thinner walls (Figure 3G–I). Hearts of dexamethasone-treated embryos showed an increase in cardiomyocyte width and volume, but not length (Figure 4A–C). Mononucleation, a marker of the cardiomyocyte ability to proliferate, was significantly decreased in dexamethasone-exposed hearts (96.0 ± 2.07 vs 85.1 ± 2.5%, *P* < .05; Figure 4D). Nuclear density was significantly decreased in dexamethasone-treated embryos (Figure 4E).

### 3.4 Markers of oxidative stress and activation of cell senescence signaling

**Figure 5:**
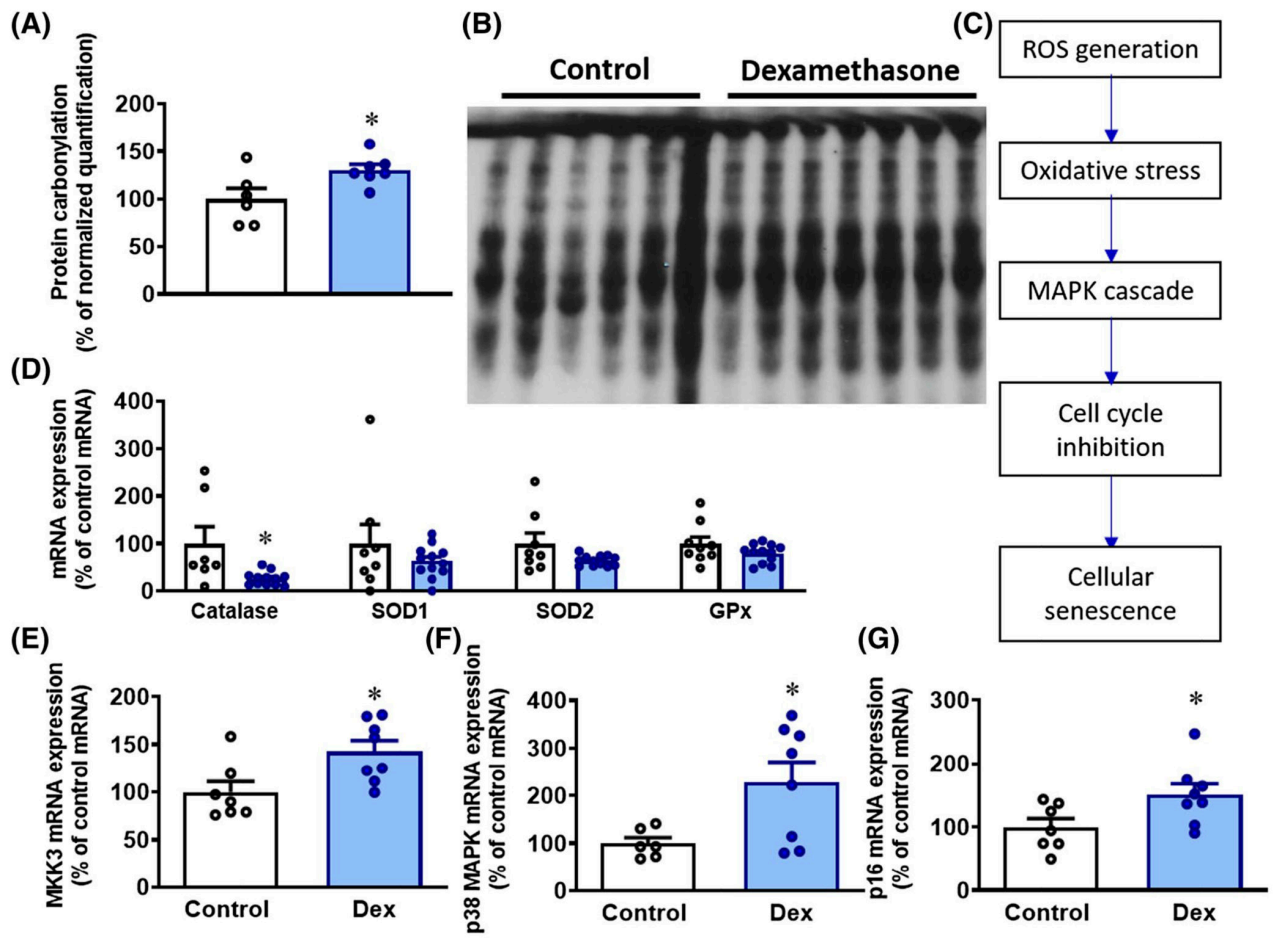
Reactive oxygen species and senescence pathways. Data are mean ± SEM. A, Protein carbonylation as compared to control samples. B, OxyBlot bands for quantification of protein carbonylation. Sample 6 was within the 2SD margin, and therefore, not excluded from the control group. C, Illustration of ROS mediated cell senescence pathway. D, mRNA expression of antioxidants Catalase, Super Oxide Dismutase 1 (SOD1), Super Oxide Dismutase 2 (SOD2), and Glutathione peroxidase (GPx), E, mRNA expression of MKK3 (also known as MAPKK3), F, mRNA expression of p38-MAPK, G, mRNA expression of p16. Significant difference *P < .05 vs control. Student’s *t* test for unpaired data

In hearts of dexamethasone-treated chicken embryos, protein carbonylation, an established marker of oxidative stress, was significantly increased, and the messenger RNA (mRNA) expression of the antioxidant enzyme catalase, but not superoxide dismutase (SOD) or glutathione peroxidase (GPx), was significantly decreased (Figure 5A,B,D). In dexamethasone-treated embryos, there was also an increase in the cardiac mRNA expression of mitogen-activated protein kinase 3 (MKK3) and its downstream kinase effector a class of mitogen-activated protein kinases (p38). Additionally, the cell cycle inhibitor a tumor suppressor (p16) was upregulated in dexamethasone-exposed hearts (Figure 5E–G).

### 3.5 Markers of cardiac fibrosis

**Figure 6:**
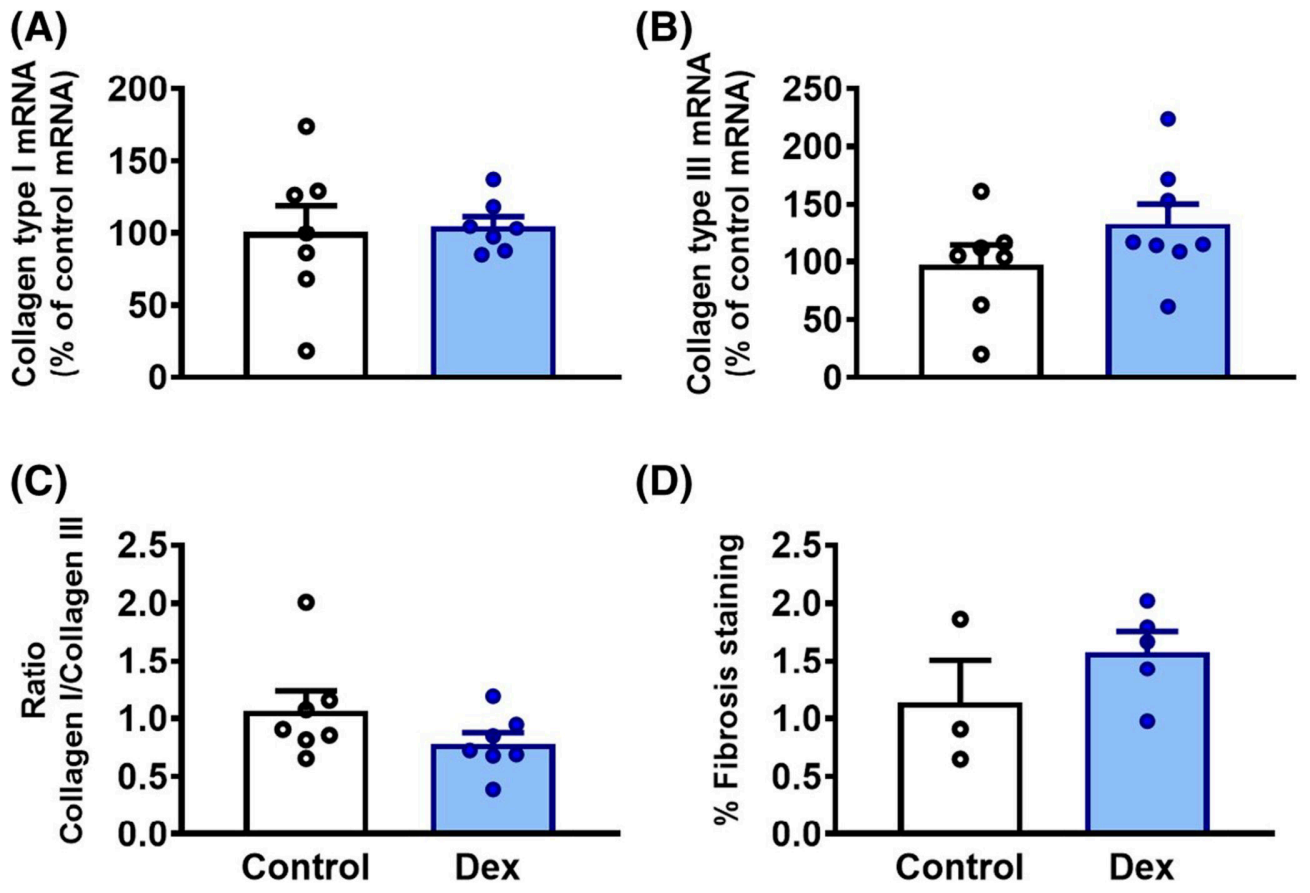
Cardiac fibrosis. Data are mean ± SEM. A, mRNA expression of collagen type I. B, mRNA expression of collagen type II. C, Ratio between mRNA expression of collagen type I and mRNA expression of collagen type III. D, Cardiac fibrosis staining with picrosirius red, expressed as a percentage of fibrotic tissue. There were no significant differences

There were no significant differences in the expression of cardiac mRNA for collagen type I or collagen type III between treatment groups (Figure 6A,B). The ratio between collagen type I and collagen type III, indicating the stiffness of connective tissue, also showed no difference between treatments (Figure 6C). There was no also difference between control and dexamethasone-treated embryos in the percentage of cardiac fibrosis staining (Figure 6D).

### 3.6 Plasma dexamethasone concentrations

Dexamethasone was undetectable in plasma from control embryos. The plasma dexamethasone concentration achieved 24 hours after treatment was 2.13 ± 0.18 nmol L^–1^; n = 7, mean ± SEM).

## 4 Discussion

**Figure 7:**
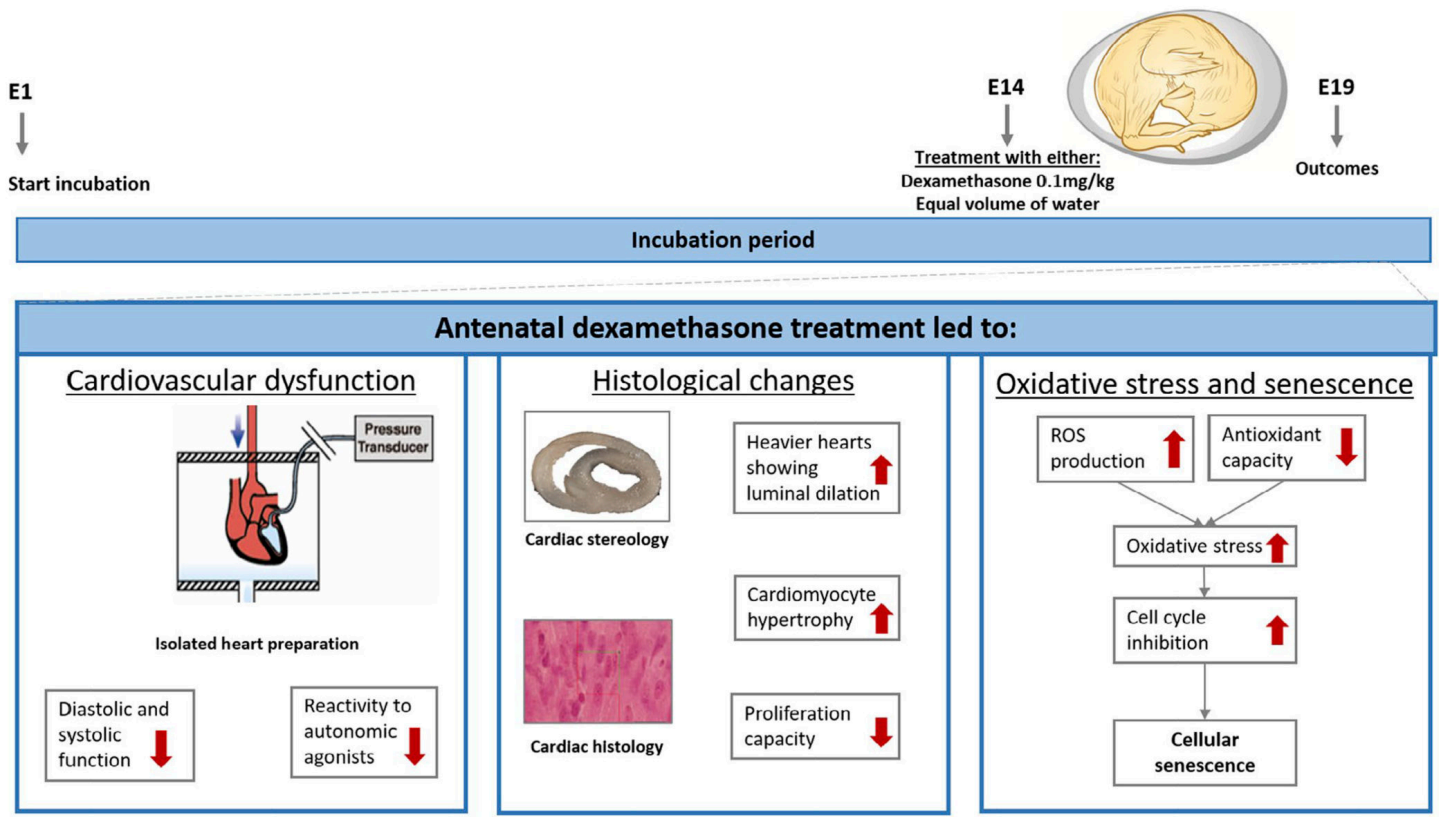
Summary figure. Chicken embryos were treated with dexamethasone or saline vehicle at embryonic day 14 (E14) and different cohorts of embryos were studied at E19. We adopted an integrative approach to study effects on cardiovascular structure and function, combining experiments at the isolated organ, cellular and molecular levels. Chicken embryos exposed to dexamethasone show systolic and diastolic dysfunction, with an increase in cardiomyocyte volume but decreased cardiomyocyte nuclear density in the left ventricle. Underlying mechanisms included increased oxidative stress, a premature switch from tissue accretion to differentiation, and activated signaling of cellular senescence

We show that direct treatment of the chicken embryo with dexamethasone at a developmental stage equivalent to the 27-week-old human fetus promotes asymmetric growth restriction and adversely affects cardiovascular structure and function. Specifically, hearts isolated from dexamethasone-treated embryos showed significant biventricular lumenal dilatation, and evidence of systolic and diastolic dysfunction. Mechanisms underlying the cardiovascular dysfunction in dexamethasone-treated embryos included blunted inotropic responses to autonomic receptor agonists, oxidative stress, cardiomyocyte hypertrophy with reduced proliferation, but no evidence of cardiac fibrosis. We propose that enhanced protein carbonylation in the heart and activation of cardiomyocyte senescence signaling pathways provides a molecular link between dexamethasone treatment and adverse cardiovascular changes in the chicken embryo (Figure 7). Therefore, the data support the hypothesis tested and provide evidence for direct adverse effects of synthetic glucocorticoids on the developing cardiovascular system independent of effects on the mother and/or placenta.

In mammalian pregnancy, the increase in fetal plasma glucocorticoid toward term is the primary maturational signal that activates many of the physiological systems that have little function in utero but are vital at birth to sustain postnatal life. Among these are respiratory, nutritive and excretory functions previously carried out by the placenta.^4,8,12^ However, the maturational effects of glucocorticoids are ubiquitous in the body and while some organ systems may benefit from exogenous glucocorticoid treatment, an early switch from tissue accretion to differentiation in other organ systems may confer potential disadvantages.

In the cardiovascular system, the prepartum increase in fetal plasma endogenous glucocorticoids stimulates the terminal differentiation of proliferative mononucleated cardiomyocytes into their binucleated form that can still undergo hypertrophy but not divide.^22^ Certainly, past data in ovine pregnancy^3,4,8,12^ and present results in the chicken embryo support that endogenous and exogenous glucocorticoids decrease the rate of fetal growth rate overall, albeit with a degree of brain sparing, leading to a reduction in body weight at term. In the heart, exogenous exposure to synthetic glucocorticoids triggers a premature fall in cell number, reducing cardiomyocyte endowment, and functional reserve capacity at term. These effects convert glucocorticoids from a maturational to a programing signal,^4,23–25^ with potential longer-term adverse consequences for cardiovascular physiological function. In the chicken embryo, all cardiomyocytes are thought to be mononucleated at hatching with binucleation starting around day 15 of post-hatching life.^26^ The current findings are consistent with this statement, showing that 96% of assessed control cardiomyocytes were mononucleated. Therefore, in the present study, treatment of the chicken embryo with dexamethasone showed evidence of an accelerated switch in the cell cycle from tissue accretion to differentiation.

This led to an earlier than normal reduction in proliferation and promoted cardiomyocyte hypertrophy already at day 19 of incubation. These effects are in keeping with an increased future risk of cardiac pathology. The end result in the present study was an increase in the presence of terminally differentiated binucleated cardiomyocytes in the dexamethasone-treated group compared to controls (13% vs 4%). While there was a reduction in cardiomyocyte nuclear density in the dexamethasone treatment group, it was not possible in this study to determine the total number of cardiomyocytes present in the left ventricle, as the hearts used to isolate cardiomyocytes, and thus, determine nuclearity were from a separate cohort from those in which cardiomyocyte nuclear density was assessed. However, the tendency toward more binucleated cells, coupled with the decreased nuclear density in the dexamethasone-treated group is in keeping with a decrease in total cardiomyocytes present in the left ventricle.

In the present study, these effects of dexamethasone modified the cardiac phenotype, yielding a weak and unresponsive heart, simulating dilated cardiomyopathy with impaired systolic and diastolic function, as well as diminished contractile responsiveness to autonomic agonists. In hearts from dexamethasone-treated embryos, the reduced contractility index is in a with an impaired inotropic responsiveness to the β_1_-adrenergic receptor agonist Isoprenaline. It is also consistent with a shortened duration of the systolic component of the cardiac cycle, although the latter effect was insufficient to lead to a significant increase in HR. The findings are of significant human clinical relevance as reduced cardiomyocyte endowment, cardiomyocyte hypertrophy, and systolic and diastolic dysfunction have all been associated with increased mortality after a cardiovascular event.^27,28^ An experiment that treated the hatchling with dexamethasone at a post-hatching timepoint similar to when the human heart starts the process of binucleation would be an interesting follow-up translational study.

Additional data in the present study support a molecular link between glucocorticoids, oxidative stress, and activated cellular senescence signaling associated with the direct deleterious effects of dexamethasone in the chicken embryo heart. Dexamethasone led to elevated oxidative damage in the heart by increasing carbonylation and reducing levels of the endogenous antioxidant catalase. In the perinatal setting, postnatal treatment of newborn rat pups with dexamethasone increased oxidative stress in the cardiovascular system, reducing NO bioavailability with adverse consequences for the cardiovascular structure and function of the offspring.^8,29–32^ In several of these studies, combined antioxidant and glucocorticoid treatment of the newborn rat pup could protect against adverse effects, underscoring a mechanistic link between dexamethasone, oxidative stress, and cardiovascular dysfunction.^8,29–34^ It is also known that increased ROS production can lead to cardiomyocyte cell-cycle arrest, and that ROS scavengers can interrupt this effect.^35^ Therefore, treatment of the chicken embryo with dexamethasone may enhance ROS production and subsequently trigger ROS-activated cellular senescence.^8,36^ In support of this idea, additional molecular data in the present study show that oxidative stress occurred with upregulation of cell cycle inhibitors, such as p16 through p38 and MKK3 as part of the mitogen-activated protein kinase (MAPK) signaling.^36,37^ The p16 pathway suppresses the expression of genes essential for proliferation, thereby promoting cell senescence.^24,38,39^ Measurement of protein expression levels and their phosphorylation status would have added to the mechanistic insight. However, given the extensive number of analyses performed within this study exhausting sample availability, and the lack of availability of validated antibodies for use with chicken tissue, this was not feasible in the current study. This is a limitation.

Direct adverse effects of dexamethasone in the chicken embryo may not necessarily occur in human pregnancy, because in mammals the placenta serves as a barrier, protecting the fetus from overexposure to excess synthetic glucocorticoids. The placental enzyme 11β-hydroxysteroid dehydrogenase (11β-HSD_2_) converts active glucocorticoids to their inactive forms.^40^ Similarly, P-glycoprotein, a multidrug resistance transporter, is expressed in the placenta where it transports glucocorticoids out of cells.^41^ However, in several species, placental expression of these two proteins declines toward term and it is known that dexamethasone is not metabolized by 11β-HSD_2_.^12^ Given that maternal treatment with synthetic steroids can impair uteroplacental perfusion,^8,11^ limiting oxygen, and nutrient transport to the fetus, the presence of the placenta in mammalian species may appear to compound rather than protect against the adverse effects of glucocorticoids on fetal cardiovascular development and function.

It is important to acknowledge that the effects of glucocorticoids on cardiovascular development not only depend upon the dose used, but also on the concentration achieved. In this study, dexamethasone treatment elevated plasma concentrations of the steroid in the chicken embryo to one-fifth of the values measured clinically^42^ in human infants and one-eighth of the values achieved in a previous study in sheep,^16^ within 24 hours after treatment. Therefore, the direct adverse effects of dexamethasone on the developing cardiovascular system of the chicken embryo occurred despite potentially lower circulating levels achieved relative to the human infant. It would be instructive to design future studies investigating dose-dependent effects of dexamethasone on the chicken embryo cardiovascular and other system. Of relevance, it is also known that metabolizing cytochrome P450 (CYP) enzymes are detectable in the chicken embryo liver from day 8 of incubation and reach peak levels at day 12 of incubation.^43^

In human obstetric practice, ACT is the same whether the pregnancy carries a male or a female fetus. Therefore, sex-dependent analysis in the present study was not an objective. However, environmental conditions during pregnancy can have sexually dimorphic effects.^44^ Therefore, from this perspective, future studies in the chicken embryo should consider effects on the female vs the male embryo. While the basic mechanism of sex determination in birds remains unknown, gonadal morphogenesis is very similar to that in mammals, and there are avian homologues for most genes implicated in mammalian sex determination.^45,46^ The cardiomyocyte data in this manuscript also mimic the changes seen in the heart of hypoxemic growth restricted sheep fetuses.^47–49^ Growth restricted fetuses are more likely to be delivered preterm, and hence are more likely to require ACT. Therefore, future studies should also consider the combined effects of hypoxemia and synthetic glucocorticoid exposure on prenatal cardiac development.

In conclusion, by simplifying the layers of complexity in mammals and employing the chicken embryo model, this study provides a significant conceptual advance to the field, isolating the direct effects of synthetic glucocorticoids on the developing cardiovascular system. We show that dexamethasone treatment in the chicken embryo promotes significant cardiac remodeling and dysfunction via mechanisms involving a premature switch from tissue accretion to differentiation, increased oxidative stress, and activated cellular senescence signaling. These direct effects of synthetic steroids on the developing offspring may contribute to the adverse effects of antenatal glucocorticoid therapy on cardiovascular function reported in early adulthood in humans.^5^ Antenatal glucocorticoid treatment of pregnant women threatened with preterm birth is here to stay, as it is life-saving therapy. However, direct adverse effects of synthetic glucocorticoids on the developing cardiovascular system need to be taken into account to fine-tune current clinical practice and make it safer for the treatment of the preterm baby.^4^

## Figures and Tables

**Table 1:** Primer sequences

Target gene	Forward primer	Reverse primer
Catalase	GCGCCCCGAACTATTATCCA	ATACGTGCGCCATAGTCAGG
Collagen type I	GATCGAGATCAGGGCCGAAG	CGAGGTCTTCGTCGTCTTGT
Collagen type III	CTGGAAGGGCAGGGAACAAC	GTACATCCTCCTAGGGCGTC
GPx	AGTACATCATCTGGTCGCCG	CTCGATGTCGTCCTGCAGTT
MKK3	CTACTTGGTGGACTCGGTGG	CGACTTCACGTTGTAGCCCT
p16	GAAGCGCGGAAGAAGACACC	GGCAACCGACGGAATGTTTG
p38MAPK	CTACTCCCCCTGCCACTTTTT	GTACACACCAGCCACCTACA
SOD1	CACGGTGGACCAAAAGATGC	GATGCAGTGTGGTCCGGTAA
SOD2	CCTGCCTTACGACTATGGCG	GCCAGCGCCTCTTTGTATTT

Note: Forward and reverse primer sequences for qRT-PCR.

Abbreviations: GPx, Glutathione peroxidase; MAPK, mitogen-activated protein kinase; MKK3, mitogen-activated protein kinase kinase 3; SOD1, Superoxide dismutase 1; SOD2, Superoxide dismutase 2.

## Abbreviations

2^-ΔΔCT^: method of presenting changes in level of gene expression
11β-HSD_2_: 11β-Hydroxysteroid dehydrogenase 2
a/p: the area that each point represents
ACT: antenatal corticosteroid therapy
AWERB: animal welfare and ethical review board
β_1_: beta 1
BSA: bovine serum albumin
C: control
*ca*.: circa
CaCl_2_·2H_2_O: calcium chloride dihydrate
CAST: computer-assisted stereological toolbox
Dex: dexamethasone
DNP: dinitrophenyl-hydrazone
GPx: glutathione peroxidase
H: the z-height of the dissector
HEPES: a zwitterionic organic chemical buffering agent
HPRT: hypoxanthine phosphoribosyltransferase
HR: heart rate
IgG: immunoglobulin G
KCl: potassium chloride
KH_2_PO_4_: potassium dihydrogen sulfate
kPa: kilopascal
LVEDP: left ventricular end diastolic pressure
LVSP: left ventricular systolic pressure
M: molar
MAPK: mitogen-activated protein kinase
MgCl_2_: magnesium chloride
MgSO_2_: magnesium sulfate
miRNeasy: kit that enables purification of total RNA
MKK3: mitogen-activated protein kinase kinase 3
mRNA: messenger RNA
NaCl: sodium chloride
NaHCO3: sodium bicarbonate
NIH: national institutes of health
p16: a tumor suppressor
p38: a class of mitogen-activated protein kinases
PFA: paraformaldehyde
Q: total number of nuclei counted
qRT-PCR: quantitative reverse transcription polymerase chain reaction
RNA: ribonucleic acid
Sdha: succinate dehydrogenase complex flavoprotein subunit A
SEM: standard error of the mean
SOD: superoxide dismutase
TBS: tris-buffered saline
TBS-T: tris-buffered saline and polysorbate
vs: versus
w/v: weight for volume
ΣP: the sum of points that lie over tissue
